# Supplemental oxygen delivery to suspected stroke patients in pre hospital and emergency department settings

**DOI:** 10.1186/2045-9912-4-16

**Published:** 2014-10-27

**Authors:** Yu-Feng Yvonne Chan, Maya Katz, Ari Moskowitz, Steven R Levine, Lynne D Richardson, Stanley Tuhrim, Kevin Chason, Kelly Barsan- Silverman, Aneesh Singhal

**Affiliations:** 1 Department of Emergency Medicine, Institute for Genomics and Multiscale Biology, Icahn School of Medicine at Mount Sinai, One Gustave L. Levy Place, 19 East 98th Street, 3rd Floor, New York, NY 10029, USA; 2 Department of Neurology, University of California, San Francisco (UCSF) Medical Center, 1635 Divisadero Street, Suite 520, San Francisco, CA 94115, USA; 3 Department of Medicine, Division of Pulmonary and Critical Care Medicine, Massachusetts General Hospital, 55 Fruit Street, Boston, MA 02114, USA; 4 Department of Neurology, State University of New York (SUNY) Downstate Medical Center, 450 Clarkson Avenue, Brooklyn, NY 11203, USA; 5 Department of Neurology, Icahn School of Medicine at Mount Sinai, One Gustave L. Levy Place, New York, NY 10029, USA; 6 Department of Neurology, Massachusetts General Hospital, 55 Fruit Street, Boston, MA 02114, USA; 7 Personalized Medicine and Digital Health, Institute for Genomics and Multiscale Biology, Icahn School of Medicine at Mount Sinai, 19 East 98th Street, 3rd Floor, New York, NY 10029, USA; 8 Genetics and Genomic Sciences, Institute for Genomics and Multiscale Biology, Icahn School of Medicine at Mount Sinai, 19 East 98th Street, 3rd Floor, New York, NY 10029, USA

## Abstract

**Background:**

Recent data suggests that high-flow oxygen started promptly after stroke symptom onset salvages ischemic brain tissue. We investigated the consistency of oxygen delivery to suspected stroke patients in the pre-hospital (PH) and Emergency Department (ED) settings, and associated adverse events (AEs).

**Methods:**

We retrospectively reviewed pre-hospital call reports of suspected stroke patients transported by our institution’s paramedics. We extracted data on oxygen delivery in the PH and ED settings, demographics, Glasgow Coma Scale score (GCS), final diagnosis, and selected AEs (mortality, seizures, worsening neurological status, new infarction, and post-ischemic hemorrhage). Patients were grouped according to ED oxygen delivery: none, low-flow (2-4 L/min), and high-flow (10-15 L/min).

**Results:**

Oxygen delivery was documented in 84% of 366 stroke transports, with 98% receiving 10-15 L/min. Our hospital received 164 patients. Oxygen delivery in the ED was documented in 150 patients, with 38% receiving none, 47% low-flow, and 15% high-flow oxygen. There were no instances of oxygen refusal, premature termination, or technical difficulties. Advanced age and low GCS predicted the use of higher flow rates. High-flow oxygen was more frequently administered to patients with symptom onset < 3 hours, and those with intracerebral hemorrhage (ICH), hypoxic-ischemic encephalopathy (HIE) or seizures (p < 0.001). More patients receiving high-flow oxygen were documented to have an AE (p = 0.02), however the low- and no-oxygen groups more frequently had multiple AEs (p = 0.01). The occurrence of AEs was predicted by the diagnosis of ICH/HIE/seizures (p = 0.013) and acute ischemic stroke (AIS)/transient ischemic attack (TIA) (p = 0.009), but not by the amount of oxygen.

**Conclusions:**

Suspected stroke patients routinely receive 10–15 L/min oxygen in the ambulance however in the ED there is wide variability due to factors such as clinical severity. Oxygen delivery appears safe in the PH and ED settings.

## Introduction

Patients with suspected stroke usually receive supplemental oxygen in the pre-hospital (PH) and emergency department (ED) settings, and oxygen is often continued after hospital admission. The dose and duration of oxygen delivery can vary in each of these clinical settings. The reasons for this variability are not clear, but in part may be explained by differences in pre-hospital and in-hospital guidelines for oxygen delivery. Paramedics favor oxygen delivery and most EMS Guidelines recommend administering high flow rates (10-15 L/min) regardless of physiologic need. Conversely, the latest AHA Stroke Treatment Guidelines [1] recommend supplemental oxygen in acute stroke only if required to maintain peripheral oxygen saturation (SaO2) levels above 92%. The evidence basis for these guidelines is relatively lacking; indeed, the threshold SaO2 level below which supplemental oxygen is recommended by the AHA has varied from 92% to 95% over the last decade.

The risks and benefits of oxygen delivery in stroke have been the subject of discussion for several decades [2-5]. Interest in a therapeutic role of oxygen in acute ischemic stroke (AIS) has recently been kindled by the results of over 120 animal studies [2-8] and pilot human studies [9-11] suggesting that the delivery of high-flow oxygen promptly after symptom onset (normobaric oxygen therapy, or NBO) affords neuroprotection and may have therapeutic potential by increasing the 3-hour time window for thrombolysis with intravenous tissue plasminogen activator (IV tPA). Our group and others have previously published and demonstrated that NBO can “freeze” the ischemic penumbra and extend the time window for thrombolysis in rodent stroke models through hemodynamic effects, improved tissue oxygenation, and biochemical mechanisms [2,6,12-14]. These recent NBO studies suggest that a paradigm of 10-15 L/min oxygen delivery – if initiated within an appropriate time window and delivered consistently for several hours in both the PH and ED settings – may provide hypoxic brain tissue oxygen, delay ischemic cell death, afford more time for arterial recanalization to occur, and ultimately improve outcomes after stoke [3,15]. Furthermore, literature supports that treating AIS patients with short durations of NBO or low hyperbaric oxygen is very unlikely to cause oxidative stress [16,17].

In this study we investigated the consistency of oxygen delivery in the PH and ED settings and relevant adverse events in the PH, ED, and inpatient settings among patients with suspected stroke.

## Methods

### Subjects

This retrospective study was conducted at our institution (Mount Sinai Medical Center, a designated stroke center) after approval by our Human Research Committee. Our institution’s EMS service transports patients to several New York City hospitals, including ours. We searched the pre-hospital call reports for consecutive patients with a presumptive diagnosis of ‘cerebral vascular accident’ encountered by our institution's EMS service from November 2003 to October 2008. We had no additional exclusion criteria for patient selection in our study. We extracted data on patient demographics, PH oxygen delivery, and PH adverse events for all patients. For the subgroup of patients brought to our institution's ED, we collected additional data on ED oxygen delivery, the inpatient medical course (for admitted patients), and relevant AEs occurring during the ED stay or inpatient admission.

### Oxygen delivery

Pre-hospital call reports and ED documentation were reviewed for completeness regarding oxygen administration. The feasibility of supplemental oxygen delivery in the PH and ED settings was evaluated by reviewing relevant documentation of any interruptions to oxygen administration and recording the reason for interruption. Nearly all patients (98%) received high flow (10-15 L/min) oxygen in the PH setting. Hence, patients were grouped according to oxygen delivery in the ED: (1) no supplemental oxygen, (2) low-oxygen (2-4 L/min), and (3) high-oxygen (10-15 L/min). For patients with changing rates of oxygen delivery, group assignment was based on the flow rate used for the longest duration. The rate of oxygen delivery (L/min) was coded as missing if not clearly documented, even if the medical records suggested that oxygen was administered. Patients undergoing endotracheal intubation were assigned to the high-oxygen group because such patients usually receive 100% oxygen around the time of intubation and thereafter during mechanical ventilation.

### Adverse events

The following adverse events (AEs) were deemed relevant based on the known clinical adverse effects of oxygen delivery and preclinical data raising potential concern for oxygen free radical injury: in-hospital mortality, seizures, and neurological decline (new altered mental status or new or worsening focal neurological deficits). For patients with the final diagnosis of ischemic stroke or transient ischemic attack (TIA), we also collected data on post-ischemic hemorrhage (any hemorrhage on a follow-up head CT or brain MRI), and recurrent ischemic stroke (new infarct in a different arterial territory on follow-up head CT or brain MRI). We assessed the relationship between the AEs and the amount of oxygen exposure, in subgroups based upon final diagnosis and the Glascow Coma Scale (GCS) score, which reflects clinical severity and is routinely documented in the ED.

### Statistical analysis

Data analysis was performed using SPSS (SPSS Inc. version 17.0, Chicago, IL). Student t test, Chi-square test, Fisher Exact test, or analysis of variance (ANOVA) was used as appropriate. Logistic regression analysis was performed to determine independent predictors of AEs, with Odds Ratios (O.R.) and 95% confidence intervals (C.I.). A value of *P* < 0.05 was considered statistically significant.

## Results

Pre-hospital call records showed that 366 patients with suspected stroke were transported to 15 hospitals during the timeframe of this study, including 164 patients (45%) transferred to Mount Sinai Hospital’s ED and 202 (55%) to other EDs. Supplemental oxygen delivery was documented in 307 patients (84%) with 301 (98%) receiving high-flow oxygen. Documentation of PH oxygen delivery was significantly higher for transfers to Mount Sinai versus other hospitals (90% versus 79%, p = 0.003), however in both groups in whom PH oxygen delivery was documented, 98% of patients received high-flow oxygen. There was no significant difference in the mean age (69 ± 15 vs. 71 ± 15 years, p = 0.22), female gender (61% vs. 63%, p = 0.86), mean on-scene time (24.5 vs. 25.2 minutes, p = 0.28), mean transport time (4.4 vs 5.0 minutes, p = 0.19), or mean PH GCS score (13 ± 3 vs. 13 ± 3, p = 0.86) between the group of patients transported to Mount Sinai versus other hospitals. Among the 164 Mount Sinai transports, the time of symptom onset or last seen well was documented in 107 patients (65%), of whom 62 (58%) arrived within the 3-hour time window for IV t-PA approved by the U.S. Food and Drug Administration. The rest arrived between 3–24 hours (22 patients, 21%) or >24 hours after symptom onset (23 patients, 21%).

Oxygen delivery status in the ED was documented in 150 patients (91%). These patients formed the denominator for subsequent analysis. PH oxygen delivery was documented in 134 patients (89%; 133 received high-flow oxygen and one received 2 L/min). In the ED, 57 (38%) received no supplemental oxygen, 70 (47%) received low-flow oxygen, and 23 (15%) received high-flow oxygen. We found no instances of patient or caregiver refusal of oxygen, disruption, premature termination, or logistical or technical difficulties related to oxygen delivery in the PH or ED settings.

Table 1 shows the characteristics of the 150 ED patients according to the category of oxygen delivery. There were significant differences in the mean age, onset to ED arrival times, initial GCS score, hospital admission status and intubation status between the 3 oxygen delivery groups. High-flow oxygen delivery tended to be common in women and those with worse GCS scores. Furthermore, of the 107 patients with documented time of symptom onset, the 62 that arrived to the ED within 3 hours of onset were more likely to receive high-flow oxygen than those presenting >3 hours or had unknown time of onset. Logistic regression analysis showed that advanced age (O.R. 1.02, 95% C.I. 1.00-1.06, p = 0.045) and poor GCS (O.R. 0.09, 95% C.I. 0.01-0.7, p = 0.02) but not sex (O.R. 1.3, 95% C.I. 0.6-2.8, p = 0.46) predicted the amount of oxygen delivery.

**Table 1:** Patient characteristics

	**No suppl. oxygen (n=57)**	**Low-flow oxygen (n=70)**	**High flow oxygen (n=23)**	**P value**
Age	65.3±15.6	73.6±13.3	65.5±16.5	0.003
Males	26 (46%)	25 (36%)	4 (17%)	0.06
Symptom onset to ED time				
Less than 3 hrs	17 (30%)	30 (43%)	11 (48%)	
More than 3 hrs	25 (44%)	17 (24%)	2 (9%)	0.02
Unknown	15 (26%)	23 (33%)	10 (43%)	
Initial GCS score				
0-8	1 (2%)	6 (9%)	8 (35%)	
9-15	53 (93%)	57 (81%)	12 (52%)	<0.001
Missing	3 (5%)	7 (10%)	3 (13%)	
Inpatient admission	44 (77%)	67 (96%)	22 (96%)	0.002
Intubation	0 (0%)	0 (0%)	17 (74%)	<0.001
Discharge destination				
Home	25 (44%)	24 (34%)	3 (13%)	
Rehab/Dead	22 (39%)	33 (47%)	15 (65%)	0.13
Unknown	10 (18%)	13 (19%)	5 (22%)	
Final Diagnosis				
AIS or TIA	22 (39%)	35 (50%)	3 (13%)	
Hemorrhagic stroke	2 (4%)	6 (9%)	8 (35%)	
HIE or Seizures	4 (7%)	4 (6%)	8 (35%)	<0.001
Toxic/metabolic	18 (32%)	17 (24%)	3 (13%)	
Miscellaneous	11 (19%)	8 (11%)	1 (4%)	
Adverse Events*				
None	43 (75%)	46 (66%)	10 (44%)	
Present	6 (11%)	13 (19%)	8 (35%)	0.07
Unknown	8 (14%)	11 (16%)	5 (22%)	
Specific Adverse Events				
Mortality	2 (4%)	5 (7%)	4 (17%)	0.2
Seizures	2 (4%)	4 (6%)	1 (4%)	0.9
New neurological deficit	4 (7%)	6 (9%)	2 (9%)	0.9
New infarction	1 (2%)	1 (1%)	1 (4%)	0.8
Post-ischemic hemorrhage	2 (4%)	6 (9%)	1 (4%)	0.7

*None of the adverse events occurred in patients discharged from the ED.

*Abbreviations:ED* emergency department, *GCS* Glasgow Coma Scale, *AIS* acute ischemic stroke, *TIA* transient ischemic attack, *HIE* hypoxic-ischemic encephalopathy.

Most patients receiving low or high dose oxygen were eventually admitted to the hospital. The final diagnosis was acute ischemic stroke in 56 patients (37%, 4 received IV tPA), TIA in 4 (3%), intracerebral hemorrhage in 16 (ICH, 11%), hypoxic-ischemic encephalopathy in 5 (HIE, 3%), toxic-metabolic encephalopathy in 38 (25%), seizure in 11 (7%), and miscellaneous conditions such as Bells’ palsy, brain tumor, and multiple sclerosis in 20 patients (13%). The amount of oxygen delivered varied significantly according to the underlying diagnosis (p < 0.001) with high-flow oxygen delivery more frequent in patients with ICH and HIE or seizures (p < 0.001) and low-flow oxygen more frequent in patients with AIS or TIA (p = 0.007). Of the 4 patients who received IV tPA, one received PH oxygen only and the remaining three continued to receive low-flow oxygen in the ED. Of the AIS/TIA patients with known time of symptom onset, 21 (57%) arrived to the ED within 3 hours and 16 (43%) arrived after 3 hours of symptom onset. Seventeen patients (11%) were discharged from the ED including one with TIA. Among the 133 admitted patients, discharge destination was known in 106 (80%); 36 went home, 58 to rehab or nursing home, and 12 expired or were discharged to a hospice facility.

Given the small sample size of AIS/TIA patients who presented within the 3 hour time window, we were unable to determine the association between oxygen use and clinical outcome (AEs) in this subset of patients. Similarly, we could not determine if interaction exists between tPA and oxygen use.

Pre-defined AEs were retrieved from medical records for 126 of the 150 patients with oxygen delivery status documented in the ED, including all 17 patients discharged directly from the ED; the rest had incomplete or missing records. The percentage of patients with any AE documented tended to be higher in the high-flow oxygen group (p = 0.07), however the percentage of specific AEs such as mortality, seizures, neurological worsening, new radiological infarcts, and post-ischemic hemorrhage, were not significantly different between groups.

Table 2 shows subgroup comparisons among the 126 patients with complete AE data. Patients receiving high-flow oxygen were more frequently documented to have an AE (p = 0.02), however most patients in this group had only one AE, while patients in the low-oxygen and no supplemental oxygen groups more frequently had multiple AEs (p = 0.01). We then examined the frequency of AEs according to the final diagnosis. In the AIS/TIA population, 15 of 53 patients (28%) were documented to have AEs and there was no significant difference in the percentage of patients with AEs, or specific AEs such as mortality and neurological worsening, across the 3 oxygen delivery groups. Among patients with a final diagnosis of ICH, HIE or seizures, 10 of 28 patients (36%) were documented to have AEs with 8 patients in the high-flow oxygen group (p = 0.06). Lastly, among patients with toxic/metabolic encephalopathy or miscellaneous stroke mimics, only 2 of 45 patients (4%) were documented to have AEs including one in the no supplemental oxygen group and the other in the low-flow oxygen group.

**Table 2:** Adverse events by final diagnosis*

	**No suppl. oxygen (n=49)**	**Low-flow oxygen (n=59)**	**High flow oxygen (n=18)**	**P value**
Any AE Present	6 (12%)	13 (22%)	8 (44%)	0.02
1 AE	2 (4%)	7 (12%)	7 (39%)	
2 AEs	3 (6%)	3 (5%)	1 (6%)	0.01
3 AEs	1 (2%)	3 (5%)	0 (0%)	
Final Diagnosis				
AIS/TIA (N=53)	N=18	N=33	N=2	
Any AE present	4 (22%)	11 (33%)	0 (0%)	0.47
Mortality	1 (6%)	4 (12%)	0 (0%)	0.67
Seizures	2 (11%)	3 (9%)	0 (0%)	0.87
New neurological deficit	3 (17%)	6 (18%)	0 (0%)	0.80
New ischemic lesion	1 (6%)	1 (3%)	0 (0%)	0.87
Post-ischemic hemorrhage	2 (11%)	6 (18%)	0 (0%)	0.66
ICH/HIE/Seizures (N=28)	N=6	N=8	N=14	
Any AE present	1 (17%)	1 (13%)	8 (57%)	0.06
Mortality	1 (17%)	0 (0%)	4 (29%)	0.24
Seizures	0 (0%)	1 (13%)	1 (7%)	0.67
New neurological deficit	0 (0%)	0 (0%)	2 (14%)	0.34
New ischemic lesion	0 (0%)	0 (0%)	1 (7%)	0.60
Other (N=45)	N=25	N=18	N=2	
Any AE present	1 (4%)	1 (6%)	0 (0%)	0.9
Mortality	0 (0%)	1 (6%)	0 (0%)	0.5
New neurological deficit	1 (4%)	0 (0%)	0 (0%)	0.7

*Patients with missing data were excluded. *Abbreviations:AE* adverse event, *AIS* acute ischemic stroke, *TIA* transient ischemic attack, *ICH* hemorrhagic stroke, *HIE* hypoxic-ischemic encephalopathy, *GCS* Glasgow Coma Scale.

Logistic regression analysis showed that AE occurrence was independently predicted by the final diagnosis of ICH/HIE/seizures (O.R. 17.3, 95% C.I. 1.84-162.7, p = 0.013), and AIS/TIA (O.R. 16.2, 95% C.I. 2.0-132.3, p = 0.009), but not the oxygen delivery category (O.R. 1.6, 95% C.I. 0.7-3.7, p = 0.23) or the GCS category (O.R. 0.8, 95% C.I. 0.7-1.05, p = 0.12). Linear regression analysis showed that the number of AEs was significantly associated with the final diagnosis of AIS/TIA (p < 0.001) and tended to be associated with the GCS category (p = 0.08) but not the final diagnosis of ICH/HIE/seizures (p = 0.12) or the oxygen delivery category (p = 0.66).

## Discussion

In this study, we assessed the amount and consistency of oxygen delivery in the initial hours after the onset of stroke-like symptoms, and its effect on immediate and delayed in-hospital adverse events. The motivation for this study comes from the overwhelmingly positive results of several rodent and pilot human studies showing that high concentrations of oxygen administered soon after ischemic stroke onset has potential to slow down the process of ischemic necrosis [2-11]. Hyperoxia’s pluripotent effects on molecular and biochemical pathways of ischemic neuronal death have been associated with reduced pathological infarct volumes, transient reversal of diffusion-MRI abnormalities, improved lactate levels, improved cerebral perfusion, and improved functional neurological outcomes. While these studies have not raised safety concerns, there is a theoretical risk of worsening stroke outcome by hyperoxia-induced oxygen free radical injury in hyperbaric oxygen treatment, cerebral vasoconstriction, and systemic adverse effects such as infection and respiratory depression [18]. Clinical trials are therefore warranted to determine whether early hyperoxia (continuous PH and ED oxygen therapy for a few hours) can safely extend the time window for thrombolysis, or improve the safety and efficacy of neuroprotective drugs by preventing early neuronal and endothelial cell death. The results of our study provide important information towards the design of such trials, which can impact stroke care around the world.

Given our institution’s location in the NYC regional EMS area, with established PH and ED oxygen delivery guidelines [19,20], we had a unique opportunity to explore the use of oxygen in the PH and ED settings in ischemic stroke as well as stroke mimics. The high frequency of oxygen administration and documentation of oxygen use in the PH setting demonstrates compliance with our PH oxygen delivery guidelines. Patients with symptom onset <3 hours or unknown time of onset more often received high-flow oxygen. While oxygen delivery status was documented in 91% of patients in our ED, there was variability in the amount delivered, reflecting biases and uncertainties in continuing oxygen after hospital arrival. Our data shows that patients who appear more ill, i.e. advanced age, low GCS, are more likely to receive higher flow rates of oxygen after ED arrival, but the flow rates selected were still variable. Nearly all patients who continued to receive oxygen in the ED were admitted, again suggesting that oxygen delivery is a marker for disease severity. Unfortunately we did not investigate the duration and amount of oxygen delivery after hospitalization. In a previous stroke study [21], approximately two-thirds received oxygen during hospitalization however there was limited justification for either giving or withholding oxygen. These data suggest opportunities to refine and improve compliance with ED and in-hospital oxygen delivery guidelines in acute stroke and other conditions [22].

In the acute setting, it can be challenging to obtain accurate information concerning chronic obstructive pulmonary disease, a relative contraindication for oxygen delivery. In our study no patient developed respiratory depression from oxygen delivery and there were no instances of refusal or technical difficulties with oxygen delivery. No adverse event was recorded among the patients discharged from the ED. While these data suggest safety and feasibility of the initial few minutes of oxygen delivery in the PH setting, it is conceivable that our paramedics withheld oxygen in patients with known COPD. Caution is still required when administering oxygen in the PH setting.

Next, we investigated the associated between oxygen delivery and subsequent adverse events. As noted, AEs were recorded solely among inpatients and not among ED discharges. Among patients with known AE status, the high-flow oxygen group had a significantly higher frequency of developing one or more AEs however patients receiving low-flow or no supplemental oxygen more often had multiple AEs. The group of patients with a final diagnosis of ICH, HIE, or seizures, had the highest frequency of AEs (36%), and patients with these diagnosis typically have more dramatic presentations, consistent with the above results showing a correlation between advanced age and lower GCS with oxygen delivery. Importantly, there was no significant difference in the occurrence of individual AEs within the final diagnosis categories (Table 2). There was no ‘pattern’ of AEs, for example higher mortality of seizures, across diagnostic groups. Only 4 of 45 patients in the stroke mimic group were recorded to have AEs, and none received high-flow oxygen. Finally, the oxygen delivery category did not prove to be a predictor of AEs in logistic and linear regression analyses. These data support the hypothesis that AEs correlate to the underlying disease process rather than the quantity of oxygen delivery. Since oxygen therapy may not be related to outcome, then our data also supports the safety of oxygen delivery in suspected acute stroke and ischemic stroke mimics. Furthermore, if the initial GCS score was a reliable indicator of disease severity, our data suggest that high-flow oxygen may potentially have a therapeutic effect. Specifically, although the percentage of HF patients (35%) with initial GCS ≤ 8 was **seventeen** times greater than that of LF (2%), the percentage of HF patients (65%) that had a discharge destination of rehab or dead was less than twice of that of LF (39%).

Although ours is the first study investigating compliance, feasibility and safety of PH and ED oxygen delivery in acute stroke, we acknowledge several shortcomings. The study was small, retrospective, single center, and limited in data collection. We did not collect information on the duration of oxygen, and hence could not adjust for the total ‘dose’ of oxygen administered. Documentation of oxygen delivery may not have been accurate in all cases. We restricted our analysis to AEs considered relevant and likely to be documented in clinical notes. Although hyperoxia can cause respiratory depression, we excluded respiratory AEs since it was difficult to determine whether oxygen caused such AEs or was administered to treat respiratory AEs (significant confounding by indication). Factors such as stroke etiology, infarct or hemorrhage volume, and co-morbidities, which contribute to in-hospital AEs, were not examined. Nevertheless, our results provide some insights and preliminary data concerning the safety and use of oxygen in PH and ED settings.

Additional limitations to our study include the lack of randomization and appropriately matched controls. Biases may be introduced in the comparison of the groups and the interpretation of the results. Given the various constraints, including the feasibility and ethical issues associated with a randomized control trial, we chose the retrospective cohort design for our study. In theory, it should offer a higher level of evidence/internal validity than case–control design because we can directly measure the absolute risk of and monitor the occurrence of multiple outcomes. Unfortunately, due to the small sample size, we were unable use to statistical techniques such as propensity score matching to help reduce potential bias. Also, we did not perform a power analysis. However, given the aforementioned study constraints, we were only able to conduct a retrospective review of the few years’ worth of medical records available to us. Therefore, the primary objective of our study was to establish feasibility and to describe the existing practice patterns of pre-hospital and Emergency Department supplemental oxygen delivery. Our secondary aim was to assess safety and report the adverse events observed in our cohort.

Previous human studies of hyperbaric oxygen therapy [23] and in-hospital supplemental oxygen [24] have not shown benefit, perhaps due to their small size, delayed treatment, and other trial design issues [25]. Most experts agree that a properly designed trial with oxygen initiated within a very narrow time window, in the PH and ED settings, is required to adequately test the therapeutic potential of oxygen in acute stroke. We envision our data to be useful in designing such trials, preferably multi-center, and hope that these trials commence in the near future.

## Competing interests

The authors declare that they have no competing interests. YC had the following research training support: NIH-NINDS 5T32 NS051147 and NIH-KL2 RR029885. AS had research support from NIH-NINDS: R01NS051412, P50NS051343, R21NS077442, and U10NS0867290.

## Authors’ contribution

YC was the lead author, contributed to study design, and performed the majority of statistical analysis and manuscript preparation. MK, and AM participated in study design, data collection, data analysis and manuscript preparation. SL provided expert guidance on study design and manuscript preparation. LD, ST, KC, and KB contributed to manuscript preparation. AS was the senior author and participated in study design, study oversight and manuscript preparation. All authors read and approved the final manuscript.
